# Utility of patients’ registries to gather clinical, epidemiological and molecular information

**DOI:** 10.1186/1750-1172-10-S2-O29

**Published:** 2015-11-11

**Authors:** Gaëlle Blandin, Christophe Béroud

**Affiliations:** 1 Aix Marseille Université, INSERM, GMGF UMR_S 910, 13385, Marseille, France

## 

Rare disease patient registries are indispensable tools for translating research into improved care and therapeutic solutions. During the race to identify a safe and effective treatment, they come into play at many stages of the translational research cycle: collection of mutational data, description of the disease, support for patient recruitment for clinical trials and scientific studies (such as natural history studies), collection of epidemiological data, evaluation/monitoring of the efficacy/safety of a treatment, elaboration of guidelines for diagnosis and management of the disease, etc.

In the field of rare disease, the main challenges that patient registries face are sustainability, better interoperability with the establishment of common data standards (for data collection, data quality, data security, legal and ethical issues) and support for translational collaborations to constitute large cohorts of patients. To work out these questions, the IRDiRC (International Rare Disease Research Consortium) initiative is a major force to encourage cooperation at international level. In this process, patients and families are becoming more active participants and must continue to raise their voice to drive innovation in collaboration with all stakeholders.

